# Characterization and Genome Analysis of a Phthalate Esters-Degrading Strain* Sphingobium yanoikuyae* SHJ

**DOI:** 10.1155/2018/3917054

**Published:** 2018-07-03

**Authors:** Liang Feng, Hui Liu, Dandan Cheng, Xumei Mao, Yan Wang, Zhen Wu, Qiong Wu

**Affiliations:** ^1^School of Environmental Studies, China University of Geosciences, Wuhan 430074, China; ^2^Hubei Key Laboratory Wetland Evolution & Ecological Restoration, China University of Geosciences, Wuhan 430074, China; ^3^State Key Laboratory of Biogeology and Environmental Geology, China University of Geosciences, Wuhan 430074, China

## Abstract

A bacterium capable of utilizing dimethyl phthalate (DMP), diethyl phthalate (DEP), di-*n*-butyl phthalate (DBP), and diisobuthyl phthalate (DIBP) as the sole carbon and energy source was isolated from shallow aquifer sediments. The strain was identified as* Sphingobium yanoikuyae* SHJ based on morphological characteristics, 16S rDNA gene phylogeny, and whole genome average nucleotide identity (ANI). The degradation half-life of DBP with substrate concentration of 8.5 and 50.0 mg/L by strain SHJ was 99.7 and 101.4 hours, respectively. The optimum degradation rate of DBP by SHJ was observed at 30°C and weak alkaline (pH 7.5). Genome sequence of the strain SHJ showed a circular chromosome and additional two circular plasmids with whole genome size of 5,669,383 bp and GC content of 64.23%. Functional annotation of SHJ revealed a total of 5,402 genes, with 5,183 protein-encoding genes, 143 pseudogenes, and 76 noncoding RNA genes. Based on genome annotation, 44 genes were identified to be involved in PAEs hydrolysis potentially. Besides, a region with size of about 6.9 kb comprised of seven ORFs, which is located on the smaller plasmid pSES189, was presumed to be responsible for the biodegradation of phthalate. These results provide insights into the genetic basis of DBP biodegradation in this strain.

## 1. Introduction

Phthalate esters (PAEs) are a class of refractory organic compounds which are widely used in plastics, coatings, and cosmetics [1]. These compounds are continuously released into environment during production, distribution, and waste disposal. PAEs have been detected in atmosphere [2], soil [3, 4], sediment [5, 6], surface water [7], and even groundwater [8]. They are considered as endocrine-disrupting chemicals (EDCs) and have effects on male and female reproduction, breast development and cancer, prostate cancer, neuroendocrinology thyroid, metabolism and obesity, and cardiovascular endocrinology [9]. Di-n-butylphthalate (DBP) and diethyl phthalate (DEP) belong to the family of PAEs, which are the most commonly used plasticizer in China.

Biodegradation has been considered as an efficient way for the removal of phthalate isomers and their esters [10]. Dozens of bacteria have been documented and described to be capable of degrading PAEs, such as* Sphingobium *sp.,* Gordonia *sp.,* Achromobacter *sp.,* Paenibacillus *sp.,* Methylobacillus *sp.,* Rhodococcus erythropolis*,* Pseudomonas *sp., and so on [11–16]. Bacterial degradation is efficient as these compounds are either biotransformed or mineralized completely [10]. The degradation of phthalate isomers can be by both aerobic and anaerobic routes and the rates of aerobic degradation are high compared to anaerobic degradation [10, 17–19]. In addition to bacteria, several fungi species can also degrade PAEs, including* Aspergillus niger* [20],* Fusarium oxysporum* [21],* Polyporus brumalis* [22],* Trichoderma harzianum* [23],* Neurospora sitophila*,* Saccharomyces cerevisiae* [24], and* Chlorella pyrenoidosa* [25].

In this study, a strain named SHJ capable of utilizing PAEs as the sole source of carbon and energy was isolated. The strain was identified as* Sphingobium yanoikuyae* based on characteristics of morphology and evidences from 16S rDNA and the whole genomic average nucleotide identity (ANI) analysis. To determine the potential biodegradation pathway of PAEs, the whole genome sequencing and functional annotation were performed. Various factors potentially affect the biodegradation of PAEs were also evaluated, including the initial substrate concentration, pH, temperature, and bacterial concentrations.

## 2. Materials and Methods

### 2.1. Chemicals

A standard sample of Di-n-butyl phthalate (purity 99.5%) was purchased from Tianjin HengXing Chemical Reagent Co., Ltd., and the stock solution was prepared at 2,000 mg/L concentration in acetone. All other chemicals and reagents were of analytical and chromatographic pure, without any purification or modification.

### 2.2. Isolation and Purification of the PAEs-Degrading Microorganism

Soil samples were collected from shallow aquifer sediments in JiangHan plain, Hubei, China. These samples were mixed and divided into two subsamples. One gram of subsample was mixed thoroughly with 100 ml of inorganic salt starch medium (ISS; NaCl, 1.0 g; K_2_HPO_4_·3H_2_O, 1.0 g; NH_4_Cl, 0.5 g; KH_2_PO_4_, 0.2 g; NH_4_NO_3_, 1.0 g; FeCl_3_, 0.01 g; MgSO_4_·7H_2_O, 0.4 g; CaCl_2_·2H_2_0, 0.02 g; H_2_O, 1000 ml; pH 7.5) supplemented with 2 g/l of dibutyl phthalate (DBP) in 250-ml Erlenmeyer flask as the sole source of carbon and energy. The culture was incubated at 18°C for 1 week statically avoiding from light and under anoxic condition. The culture was repeatedly for twice under the same conditions. Subsequently, 0.2 ml of the enrichment culture was spread onto ISS agar plates containing 2 g/l of DBP and incubated at 30°C for 96 hours under anoxic conditions. Individual pure colonies were then streaked in fresh ISS agar plates with or without DBP and incubated for 5 days. One pure colony which could grow on ISS agar plates with DBP and not grow on ISS agar plates without DBP was obtained and named as SHJ.

### 2.3. Bacterial Growth and Genomic DNA Preparation

Strain SHJ were grown and maintained at 37°C in NB medium broth (beef extract, 3 g; peptone, 5 g; NaCl, 5 g; H_2_O, 1000 ml; pH 7.5). For genomic DNA isolation, bacterial cells were harvested at OD_600_=0.6 by centrifugation, followed by washing twice with PBS buffer (pH 7.4). Genomic DNA was then isolated with the UltraClean® Microbial DNA Isolation Kit (MoBio) according to the manufacturer's protocol. The DNA concentration was determined using UV-vis Spectrophotometer (Nanodrop 2000, ThermoFisher Scientific, Wilmington, USA) and Quant-iT™ PicoGreen® dsDNA Assay Kit (P11496 Invitrogen™, ThermoFisher Scientific, Wilmington, USA). The quality and integrity of isolated DNA were checked by electrophoresis on a 0.8% agarose gel.

### 2.4. Single-Molecule Real-Time Sequencing

The DNA libraries were prepared following the PacBio guidelines and sequenced on a SMRT cell using Pacific Biosciences RS II sequencing technology. Briefly, 5 *μ*g of genomic DNA was mechanically sheared to an average size of 20,000 bp, using a Covaris gTube (Covaris Inc., Woburn, MA, USA) according to the manufacturer's instructions. The sheared gDNA were DNA damage repaired and end-repaired using polishing enzymes. A blunt end ligation reaction followed by exonuclease treatment was performed to create the SMRT bell template. The library was size-selected using BluePippin Size Selection System (Cat #BLU0001, Sage Science). The purified library concentrations were determined by Qubit 2.0 Fluorometer (Cat #Q32866, Life Technologies). Sequencing primers were annealed to the SMRTbell templates followed by binding with the complex using the DNA/Polymerase Binding kit P6 with the magBead loading kit (Pacific Biosciences). The final SMRTbell library was sequenced using one SMRT cell (Pacific Biosciences) with C4 chemistry (DNA sequencing reagent 4.0), taking one movie of 360 minutes using the PacBio RS II instruction.

### 2.5. Genome Assembly and Annotation

The subreads generated from the raw sequencing reads were* de novo* assembled using Hierarchical Genome Assembly Process (HGAP) version 3.0 [27]. The polished assemblies were examined for circularity based on the presence of overlapping sequences at both ends of the contig. Location of the overlapping sequence was determined using MUMmer version 3.0 [28], and one of the overlapping ends was removed to yield a complete genome map. The complete genome sequences were annotated by the Prokaryotic Genome Annotation Pipeline (http://www.ncbi.nlm.nih.gov/genomes/static/Pipeline.html).

### 2.6. Phylogenetic Analysis and Average Nucleotide Identity Calculation

16S rDNA sequences from various strains of the genus* Sphingobium* were extracted from the National Centre for Biotechnology Information (NCBI) and were aligned using MUSCLE (version 3.8.425) with the default settings [29]. The resulting alignment was used as an input in MEGA software version 7.0 [30]. The best substitution model for phylogeny inference by the maximum-likelihood (ML) method was searched by the model test option of MEGA 7.0 and the Kimura 2-parameter model with nonuniformity of evolutionary rates among sites by using a discrete Gamma distribution (+G) with five rate categories and by assuming that a certain fraction of sites are evolutionarily invariable (+I) was used for construction of a ML tree. The phylogenetic tree topology was evaluated by bootstrap analysis (1,000 replicates). Average Nucleotide Identity (ANI) was calculated using JSpecies software version 1.2.1 [31], and the result of MUMmer based calculation was selected to be shown [29].

### 2.7. Degradation of DBP by the Isolate SHJ in Pure Culture

The isolate SHJ was precultured in 500-ml Erlenmeyer flasks containing 100 ml of NB medium at 30°C and 120 rpm on a rotary shaker for 48 hours. The cells were harvested by centrifugation (4000 ×g, 10 min), washed 3 times with 10 ml of sterilized double distilled water, and finally suspended in 10 ml of sterilized saline solution. In order to better understand the biodegradation of DBP by the isolate SHJ, the mineral salts medium (MSM; K_2_HPO_4_, 0.065 g; KH_2_PO_4_, 0.0255 g; Na_2_HPO_4_·12H_2_O, 0.1338 g; NH_4_Cl, 0.0051 g; CaCl_2_, 0.0825 g; MgSO_4_·7H_2_O, 0.0675 g; FeCl_3_·6H_2_O, 0.00075 g; H_2_O, 1000 ml; pH 7.5) supplemented with DBP was used as the sole carbon and energy source. Each flask was supplemented with specified DBP concentration and incubated statically in the dark. Selection of DBP concentration was based on following considerations: (1) the concentration of DBP must be extremely higher than that in contamination water (the concentration of DBP in serious contaminated water is up to several to hundred *μ*g/L); (2) DBP is miscible with common organic solvents while almost insoluble in water (13 mg/L at 25 degrees). We prepare the stock solution with acetone. The concentration of acetone must be controlled as it might inhibit the growth of the strain; (3) biodegradation half-lives of DBP extended as the initial concentration increases. Thus we have to control the concentration of DBP so that the whole experimental period is reasonable and acceptable. DBP residues were determined after incubation for 10 days, and each treatment was performed in triplicate. To assess the effects of DBP concentrations on its biodegradation, the medium (pH 7.5, biomass level of the isolate SHJ, 1.0×10^7^ CFU/ml) was supplied with DBP at two levels of 8.5 mg/L and 50.0 mg/L. To determine the effect of temperature on the biodegradation, the culture (pH, 7.5; biomass level of the isolate SHJ, 1.0×10^7^ CFU/ml) was incubated at 12°C, 16°C, 20°C, 24°C, 28°C, 30°C, 32°C, 34°C, 36°C, and 38°C, respectively. To evaluate the effect of pH on the biodegradation, the culture (biomass level of the isolate SHJ, 1.0×10^7^ CFU/ml) was prepared with buffers at pH 5.0, 5.5, 6.0, 6.5, 6.5, 7.0, 7.5, 8.0, and 9.0, respectively. To estimate the effect of biomass level of the isolate SHJ on the biodegradation, the culture (pH, 7.5) was inoculated with a suspension of the isolate SHJ at five biomass levels of 0, 1×10^6^, 3.0×10^6^, 9.0×10^6^, and 2.7×10^7^ CFU/ml, respectively.

### 2.8. Chemical Analysis

DBP was quantified using a HPLC (Shimadzu LC-10AT, Japan) with a UV detector (SPD-10AV, Shimadzu) at 224 nm. After centrifugation at 12,000 g for 5 min, sample was separated using an Allsphere Kromasil C18 column (150×4.6 mm, particle size 5 *μ*m) (AkzoNobel, Madein). The mobile phase was a mixture of methanol and water at the ration of 80/20. The mobile phase was delivered at a flow rate of 1.0 mL/min.

### 2.9. Statistical Analyses

All the statistical analysis was performed by using statistical analysis tools SPSS 13.0 using one-way ANOVA (analysis of variance). The data was evaluated in 95% significance interval (A p < 0.05 was considered as the threshold for statistical significance).

### 2.10. Nucleotide Sequence Deposition

The whole genome shotgun project has been deposited at DDBJ/EMBJ/GenBank under the accession numbers CP020925, CP020926, and CP020927 (BioProject ID PRJNA239177 and BioSample ID SAMN02666059).

## 3. Results and Discussion

### 3.1. General Characterization of the Strain SHJ

The bacterial strain SHJ was isolated from shallow aquifer sediments in JiangHan plain, Hubei, China. The SHJ cells were short rods ranging in length and width within 0.4~0.6 *μ*m by 1.1~2.6 *μ*m. Its colony was ivory, opaque, and round, forming in 24~36 hours with diameter of 0.1~0.5 mm on NB agar at an optimal temperature of 25°C. Colonies turned to yellow when culturing time was extended to 72 hours. It is mesophilic and demonstrates a range for growth temperature from 13°C to 30°C. The isolate SHJ was Gram-negative and tested positive for L-arabinose, D-xylose, galactose, salicin, mannose, D-turanose, and caprate. The degradation of common individual PAEs, including dimethyl phthalate (DMP), diethyl phthalate (DEP), di-n-butyl phthalate (DBP), and di-isobuthyl phthalate (DIBP) was measured, and it could use either of the compounds as the sole carbon and energy sources for growth. DBP is one of the most commonly used plasticizers, as well as an additive to adhesives or printing inks. Thus, DBP was selected for studying the biodegradation kinetics of SHJ.

### 3.2. Effect of Substrate Concentration on the Biodegradation of DBP

**Figure 1:**
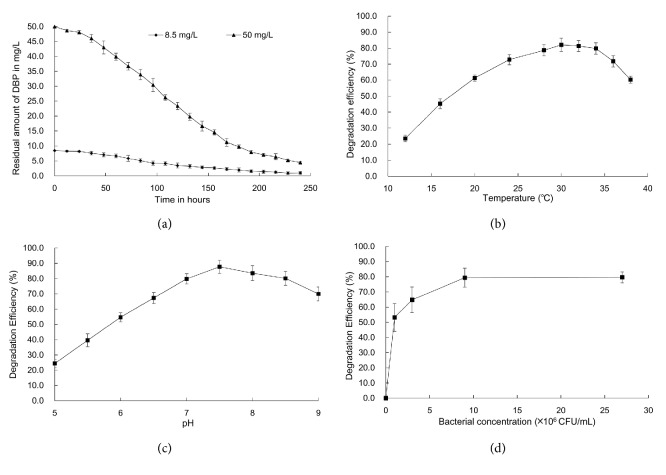
Effects of substrate concentration (a), temperature (b), pH (c), and the initial bacterial cell concentration (d) on the degradation of DBP by the isolate SHJ in mineral salts medium. Error bars indicate the standard deviations with n=3.

The biodegradation effects of different concentration of DBP by the isolate SHJ in MSM of pH 7.5 at 30°C are shown in Figure 1(a) and kinetic data are summarized in Table 1. After incubation for 10 days, the biodegradation rate of DBP in two concentrations of 8.5 and 50 mg/L was 0.0313 and 0.1867 mg/L/h, respectively. Kinetic analysis reveals that the biodegradation of DBP by SHJ was best fitted to the first-order kinetics. The biodegradation half-lives of DBP at concentrations of 8.5 and 50.0 mg/L were 99.7 and 101.4 h, respectively. Previously, facultative anaerobe bacteria were isolated and identified as* Ochrobactrum *sp. JDC-41 and the biodegradation half-lives of DBP extended from 3.83 h to 18.12 h as the initial DBP concentration increased from 50 mg/L to 500 mg/L [32].

### 3.3. Effect of Temperature on the Biodegradation of DBP

The effect of various temperatures on the biodegradation of 50.0 mg/l of DBP by the isolate SHJ in MSM at pH 7.5 is shown in Figure 1(b). The ANOVA analysis indicated that the degradation rate of DBP by strain SHJ significantly increased with temperature from 12°C to 20°C, and no significant differences were observed when temperature increased from 24°C to 36°C. The highest degradation rate of DBP was observed at 30°C. Our findings are consistent with previous report, which showed that the biodegradation rates of PAEs by* Pseudomonas fluorescences* FS1 significantly decreased when the temperature was below 10°C or above 35°C[33].

### 3.4. Effect of pH on the Biodegradation of DBP

The biodegradation of DBP (50.0 mg/l) by the isolate SHJ in MSM of pH 5.0, 5.5, 6.0, 6.5, 7.0, 7.5, 8.0, 8.5, and 9.0 is shown in Figure 1(c). Our results indicated that the alkaline condition at pH 7.5 was favorable for the degradation of DBP, and it was also the optimum pH for the growth of strain SHJ. A similar result was also reported previously, which showed that the optimum pH for PAEs biodegradation by* Acinetobacter* sp. JDC-16 is around 8.0 [34]. It was speculated that the acidic substance generated during biodegradation (such as phthalate acid) would acidify the media, which slows down the process. However, the hydroxyl ions in alkaline media could avoid the accumulation of the acidic substance through neutralization, which protect the bacteria from the toxic effect caused by acid substances [34].

### 3.5. Effect of the Initial Bacterial Cell Concentration on the Biodegradation of DBP

The effect of the initial bacterial cell concentration on the biodegradation of 50.0 mg/L of DBP by the isolate SHJ in MSM at pH 7.5 is shown in Figure 1(d). The biodegradation rate of DBP by SHJ increased from 53.2% to 79.4% when the initial bacterial cell concentration increased from 1.0×10^6^ CFU/mL to 9.0×10^6^ CFU/ml. The ANOVA analysis indicated that no significant differences were observed when the initial bacterial cell concentration increased from 9×10^6^ CFU/ml to 2.7×10^7^ CFU/ml. The bioremediation effect is improved and the time required for the process shortened when the initial bacterial cell concentration increased. However, an excessive of the initial bacterial cell concentration might hamper the biodegradation of PAEs through formation of bacterial zoogloea or production of harmful metabolites [35].

### 3.6. Genome Properties

**Figure 2:**
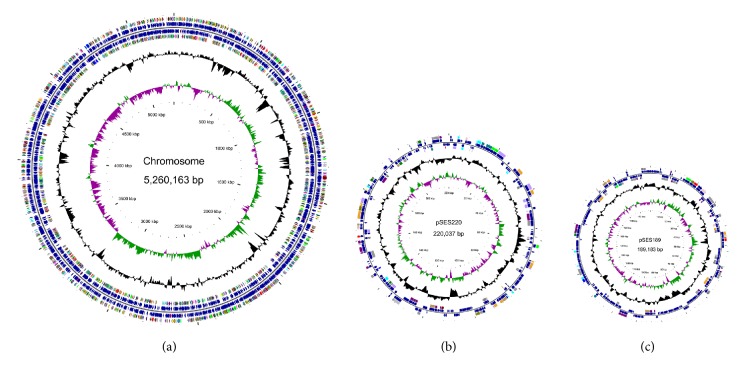
Circular representation of the three replicons of* Sphingobium yanoikuyae* strain SHJ. (a). Chromosome; (b) plasmid pSES220; (c) plasmid pSES189. Circles display from the inside to the outward, (1) circle 1: scale in kb; (2) circle 2: GC-skew (G-C/G+C ratio) using a 1 kb window with 100 bp step; (3) circle 3: GC content using a 3 kb window with 100 bp step; (4) circle 4: COG assignments for predicted CDSs on the forward strand; (5) circle 5: all genes on the forward strand; (6) circle 6: COG assignments for predicted CDSs on the reverse strand; (7) circle 7: all genes on the reverse strand. The whole genome map was generated using CGView [26].

The complete genome of SHJ contained 1 circular chromosome and 2 circular plasmids. The size of chromosome 1, plasmid pSES220, and plasmid pSES189 were 5,260,163 bp, 220,037 bp, and 189,183 bp, respectively, and summed to 5,669,383 bp. The GC content of SHJ genome was 64.23%. A total of 5,402 genes were identified, 5,183 of which were protein-coding genes and 143 were pseudogenes. The remaining 76 genes were RNA genes, including 12 rRNA genes, 61 tRNA genes, and 3 other noncoding RNA genes (Figure 2, Table 2).

**Figure 3:**
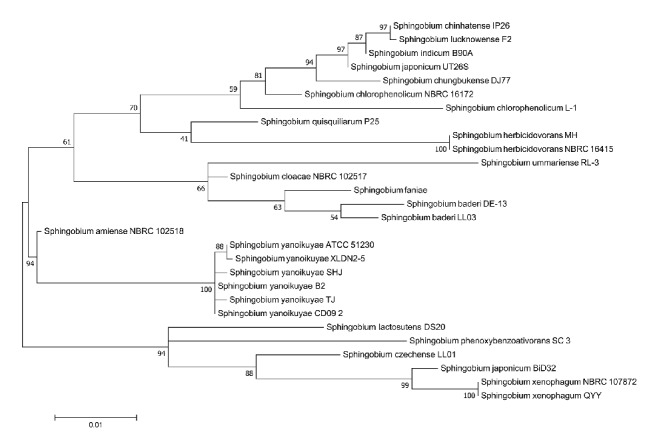
Unrooted phylogenetic tree based on 16S rDNA sequence of the genus* Sphingobium*. The scale bar represents the number of changes per sites. Numbers at branch-points are percentages of 1000 bootstrap resamplings that support the tree topology.

The 16S rDNA gene sequence (1,488 bp) of strain SHJ was searched against the GenBank database. It shows 99% identity to* Sphingobium yanoikuyae* strain B2. Phylogenetic analyses based on 16S rDNA sequences within genus* Sphingobium *suggested SHJ best fitted into the species* Sphingobium yanoikuyae *(Figure 3). Average nucleotide identity (ANI) values in the range of ≥ 95-96% correspond to ≥ 70% DDH standard for species definition [36]. Whole genome average nucleotide identity (ANI) values were obtained from pairwise comparison of the available genome sequences in the genus* Sphingobium*. ANI values between SHJ and the genus* Sphingobium* members were in the range of 84.27-96.50% and ANI values between SHJ and the species* Sphingobium yanoikuyae* members were in the range of 96.26-96.50%, surpassing the thresholds for species definition (Supplemental Table S1). Combined with morphology characterization, 16S rDNA, and ANI analysis, strain SHJ was classified as* Sphingobium yanoikuyae *strain SHJ.

### 3.7. Biodegradation Pathway of DBP Revealed by Genome Annotation

PAEs biodegradation by SHJ was observed to be an aerobic process. It was firstly hydrolyzed by an esterase to the corresponding monoesters and free phthalate [37]. We identified 2, 11, and 31 genes encoding for acylesterase, esterase/carboxylesterase, and hydrolase/alpha/beta hydrolase, respectively. 3 of them were located on plasmids and the remaining 41 of them were located on chromosome (Supplemental Table S2). 13 of these esterase and hydrolase showed low amino acid identity to those of previously reported enzymes for phthalate esters hydrolysis (Supplemental Table S3). For example, BV87_04990, BV87_19970, BV87_23980, and BV87_13955 from SHJ showed 38.27%, 34.06%, 32.52%, and 31.50% identity with carboxylesterase (accession no. AIZ00845.1) from* Bacillus *sp. K91 with 97.96%, 98.16%, 96.52%, and 97.34% coverage [38], respectively; BV87_04950 showed 31.33% identity with dialkyl PEs hydrolase (accession no. AGY55960.1) from an uncultured bacterium with 71.75% coverage [39]. We also noticed that BV87_04545, BV_20390, BV87_20075, BV87_01010, and BV87_17485 showed less than 30% amino acid identity with esterase (accession no. AEW03609.1) from* Sulfobacillus acidophilus* DSM 10332 [40] and monoethylhexylphthalate hydrolase (accession no. BAE78500.1) from* Gordonia sp. *P8219 [41], respectively, at high coverage (74.01%~92.28%). Functional validation with experiment of these genes would be beneficial for understanding the mechanism of PAEs-degradation in genus* Sphingobium*.

**Figure 4:**
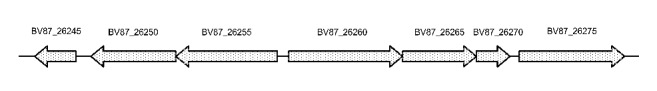
Gene organization of the potential phthalate degradation cluster in strain SHJ. The potential phthalate genes from BV87_26245 to BV87_26275 encode a MarR family transcriptional regulator, 4,5-dihydroxyphthalate decarboxylase, 4,5-dihydroxyphthalate dehydrogenase, phthalate 4,5-dioxygenase oxygenase subunit, nicotinate-nucleotide pyrophosphorylase, ferredoxin, and pyridine nucleotide-disulfide oxidoreductase.

In Gram-negative bacteria, the phthalate could be transformed to protocatechuate by phthalate catabolic gene cluster (*pht*). Whole genome analysis of strain SHJ revealed a region with size of about 6.9 kb, located on the smaller plasmid pSES189, might be involved in biodegradation of PAEs (*phtAaAcAdBCDR*). The region is comprised of seven open reading frames, which were transcribed in opposite direction (Figure 4, Table 3). Phthalate is initially transformed into 4,5-dihydro-4,5-dihydroxyphthalate by phthalate dioxygenase, a two-component enzyme consisting of phthalate dioxygenase reductase and phthalate dioxygenase oxygenase.* phtAa* (BV87_26260) encoded for phthalate dioxygenase oxygenase involved in the transformation of the phthalate into 4,5-dihydro-4,5-dihydroxyphthalate, and it showed 53% amino acid sequence identity to the phthalate 4,5-dioxygenase oxygenase subunit from* Pseudomonas putida* strain NMH102-2 (accession no. Q05183) [42]. We did not find any gene encoding for phthalate dioxygenase reductase (*phtAb*). The plant-type [2Fe-2S] ferredoxin (*phtAc*, BV87_26270) and the ferredoxin reductase (*phtAd*, BV87_26275) were identified, located about 860 bp apart from the* phtAa* gene, and in the same transcriptional orientation. The 4,5-dihydroxyphthalate dehydrogenase (*phtB*, BV87_26255) removes two electrons and two hydrogens from* cis*-phthalate dihydrodiol to form NADH and 4,5-dihydroxyphthalate. Finally, one of the two carboxyl groups of the latter compound is removed by 4,5-dihydroxyphthalate decarboxylase (*phtC*, BV87_26250) to form protocatechuate, a central metabolite in the catabolism of aromatic compounds.* phtD* (BV87_26265) coding for quinolinate phosphoribosyl transferase, which enhances the ability of Strain DBO1 to grow on phthalate while not being required for the actual metabolism of phthalate [43]. The MarR family transcriptional regulator (*phtR*, BV87_26245) participates in the regulation of phthalate degradation.

## 4. Conclusions

A* S. yanoikuyae* strain SHJ was able to utilize DMP, DEP, DBP, and DIBP as the sole carbon and energy source. The optimum degradation rate of DBP by SHJ was observed at 30°C and weak alkaline (pH 7.5). Genome analysis revealed that multiple genes probably are involved in biodegradation of DBP. Our results provides the groundwork for further elucidation of the genetic basis of DBP degradation in strain SHJ.

## Figures and Tables

**Table 1:** Biodegradation kinetic data of DBP by the isolate SHJ at different substrate concentrations in MSM of pH 7.5 at 30°C. t_1/2_: the degradation half-lives of DBP.

**Concentration**	**Kinetic function**	**Degradation rate**	**t**_**1/2**_	**r** ^**2**^
**(mg/L)**		**(mg/L/h)**	**(h)**	
8.5	ln *C* = -0.01t+2.4441	0.0313	99.7	0.9915
50.0	ln *C* = -0.0116t+4.3952	0.1867	101.4	0.9804

**Table 2:** General features of the *S. yanoikuyae *SHJ genome.

	**Chromosome**	**pSES220**	**pSES189**	**Genome**
**Size (bp)**	5,260,163	220,037	189,183	5,669,383
**GC content**	64.35	63.69	61.42	64.23
**Total genes**	4,990	222	190	5,402
**tRNA genes**	61	0	0	61
**rRNA genes**	12	0	0	12
**Other non-coding RNAs**	3	0	0	3
**Pseudo genes**	118	13	12	143
**Total number of CDSs**	4,796	209	178	5,183
**CDSs with assigned function**	3,538	126	111	3,775
**Hypothetical proteins**	1,258	83	67	1,408
**CDSs assigned to COGs**	3,250	117	85	3,452
**CDSs assigned to KEGG Ontoloty**	1,213	16	8	1,237
**CDSs assigned to GO function**	2,536	82	83	2,701
**CDSs with Pfam domains**	3,880	147	133	4,160
**CDSs with signal peptides**	674	10	15	699
**CDSs with transmembrane helices**	1,056	39	36	1,131

**Table 3:** Potential genes involved in biodegradation of phthalate.

**Locus tag**	**Gene symbol**	**Length (bp)**	**Description**
BV87_26245	*phtR*	483	MarR family transcriptional regulator
BV87_26250	*phtC*	993	4,5-dihydroxyphthalate decarboxylase
BV87_26255	*phtB*	1,188	4,5-dihydroxyphthalate dehydrogenase
BV87_26260	*phtAa*	1,335	Phthalate 4,5-dioxygenase oxygenase subunit
BV87_26265	*phtD*	864	nicotinate-nucleotide pyrophosphorylase
BV87_26270	*phtAc*	399	2Fe-2S ferredoxin
BV87_26275	*phtAd*	1,233	Ferredoxin--NAD(P)(+) reductase fdr

## Data Availability

The data used to support the findings of this study are available from the corresponding author upon request.

## Supplementary Materials

Supplementary 1 Table S1. Whole genome average nucleotide identity (ANI) analysis of* Sphingobium yanoikuyae* strain SHJ with other strains from genus* Sphingobium*.

Supplementary 2 Table S2. Potential esterase and hydrolase genes identified in* Sphingobium yanoikuyae* strain SHJ.

Supplementary 3 Table S3. Best hits of potential esterase and hydrolase proteins against nr database.
